# The Efficacy and Biopharmaceutical Properties of a Fixed-Dose Combination of Disulfiram and Benzyl Benzoate

**DOI:** 10.3390/ijms231810969

**Published:** 2022-09-19

**Authors:** Maria Lajarin-Reinares, Elia Martinez-Esteve, Eloy Pena-Rodríguez, Mariona Cañellas-Santos, Sanja Bulut, Kostas Karabelas, Adam Clauss, Carles Nieto, Mireia Mallandrich, Francisco Fernandez-Campos

**Affiliations:** 1 Department of Genetics and Microbiology, Campus Microbiology Unit, Autonomous Barcelona University, 08193 Barcelona, Spain; 2 Topical & Oral Development, Research and Development Reig Jofre Laboratories, 08970 Barcelona, Spain; 3 Biotechnology Department, Research and Development, Reig Jofre Laboratories, 08970 Barcelona, Spain; 4 Department of Development, Bioglan AB, 21124 Malmö, Sweden; 5 Department of Pharmacy, Pharmaceutical Technology and Physical Chemistry, School of Pharmacy and Food Sciences, University of Barcelona, 08028 Barcelona, Spain

**Keywords:** disulfiram, benzyl benzoate, skin permeation, topical medication, antiparasitic agents, scabies, lice

## Abstract

Scabies and hair lice are parasitic diseases that affect human skin and hair, respectively. The incidence and resistances of these infections are increasing. Tenutex^®^ (disulfiram and benzyl benzoate emulsion) is an alternative to standard insecticides to avoid resistances. The aim of the work is to evaluate the transdermal absorption and the in vitro efficacy against scabies and hair lice after different exposition times. Dermatomed human skin was used to assess the dermal absorption using a validated High Performance Liquid Chromatography (HPLC) method. HEK001 keratinocytes were used to evaluate the cytotoxicity of benzyl benzoate. Only benzyl benzoate was able to cross the skin, but it did not show cytotoxicity at any of the tested concentrations. The product efficacy was tested on *Psoroptes ovis* after direct contact and after administration on sheep skin explants at different contact times. Permethrin/malathion-resistant strains of *Pediculus humanis capitis* adults and eggs were directly exposed to Tenutex, and the vitality and hatchability, respectively, were evaluated. The anti-scabies study demonstrated that exposure for 6 or 24 h completely eradicated the parasite. The pediculicidal activity of Tenutex exhibited superior efficacy than standard treatment on resistant lice. The positive results obtained suggest that Tenutex^®^ is a good treatment option, especially in drug resistance situations.

## 1. Introduction

Scabies is a superficial skin disease caused by *Sarcoptes scabiei* mites. It is a world-wide infection, estimated to affect from 130 million [1] to 300 million [2] people per year. Other authors estimated the global prevalence at 0.2–70%, depending on the geographical location [3]. It spreads easily in crowded areas (including elderly care homes and military environment) and those with poor sanitation; due to migration pressure, cases are increasing in developed countries [4]. The parasites produce tunnels under the skin with erythematous and pruritic lesions, which could present as pustules, vesicles, and nodules. Furthermore, scratching of pruritic lesions can lead to bacterial infection (*Staphylococcus aureus* and *Streptococcus pyogenes*), which further worsens the pathology [3]. Skin symptoms usually appear four weeks after the primary infection, together with unspecific signs (discomfort, eruptions, eczema, etc.) common to other skin diseases. Furthermore, the frequent low number of adult parasites present on the skin makes diagnosis difficult; microscopic observation of parasite eggs and/or feces is usually required [5]. *Sarcoptes* are usually observed in skin folds (i.e., fingers, arms, wrist, genital area, axillae) and around the trunk in general. The neck and face are not usually affected in adults but could be affected in infants [6]. The entire life cycle of the mite occurs in humans. Adult individuals mate on the skin surface and females lay their eggs in skin burrows within the stratum corneum. After 2–3 weeks, eggs hatch into larvae that after several days transform into nymphs (with two developmental stages, the protonymph and tritonymph) that will eventually become adults. The development time from egg to adult can be up to 21 days, and the adult life span ranges from 26 to 40 days [7]. 

Several hygiene measures should be considered to reduce the recurrence and spread of the infection, e.g., the treatment of sexual partners and washing clothes and bedsheets at high temperatures [6]. Apart from these precautions, there are several pharmacological treatments to eradicate mites, generally insecticides that are given either topically or orally. Recommended treatments, according to the European Academy of Dermatology and Venereology [8], include topical permethrin (5% cream) or benzyl benzoate lotion (10–25%), or oral treatment with ivermectin. As an alternative treatment, the topical administration of malathion, ivermectin, sulfur, or synergized pyrethrin has been suggested. Other remedies to treat scabies include topical treatment with crotamiton, monosulfiram, and lindane. The more severe form of infestation, crusted scabies, occurring mainly in immune-compromised patients, requires a combination of topical scabicides and oral ivermectin. The gold standard of pharmacological treatment of scabies is topical dosage forms with 5% permethrin. Treatment usually consists of a single dose, covering the entire body surface except the face. It is a multidose regimen, i.e., one dose per day for five days, or two single doses separated by one week, obtaining, in most cases, the complete eradication of the disease and very good tolerance [3]. 

Drug resistance has not been widely studied; however, it has been described in literature [7]. There is increasing evidence of drug resistance and several case studies report lower-than-expected topical permethrin effectiveness [9,10,11,12,13]. Thus, the availability of alternative treatments is of increasing concern.

*Pediculus humanus* var. *capitis*, commonly known as hair lice, is an ectoparasite that infests the human scalp. It feeds from blood from the infestation site. Female lice produce eggs that attach to the hair and hatch in approximately one week. Within 7–10 days after hatching, the nymph becomes an adult that can live up to approximately 30 days [14]. Pediculosis, or infestation by *Pediculus humanus*, usually affects up to 13% of children 5–13 years old. Although it is not a severe disease, it is bothersome and can cause irritation, itching, social rejection, school access limitations, and a secondary skin infection due to scratching—for example, pyoderma by Streptococcus or Staphylococcus spp. [15]. The main principles for the treatment of head lice include the use of noninsecticide agents, insecticide agents, and wet combing. In many European countries, the use of noninsecticide agents has become the treatment of choice and has overtaken insecticide-based products, probably due to safety concerns and increasing drug resistance [16]. Noninsecticide pediculicides have a physical mode of action, coating the lice and causing them to die via suffocation and/or dehydration. Most noninsecticide products contain either synthetic silicon oils, such as dimeticones, or isopropyl myristate or 1,2-octanediol as active pharmaceutical ingredients (APIs) [17]. 

The heavy use of neurotoxic insecticides as pediculicides has led to the emergence and spread of drug resistance in many parts of the world [18]. For example, resistance to permethrin has been reported, and the success rate of permethrin as a pediculicide in some parts of the UK is only 30–40%. The reported frequency of permethrin-resistant head lice is extremely high in Denmark [19,20]. Resistance to malathion has been reported in Denmark, France, the UK, and Australia, although resistance to malathion seems to be less common compared to pyrethroids [20,21,22,23]. Double resistance to permethrin and malathion in head lice has also been documented among school children in the UK [22]. Resistance to lindane is well known from many countries and has been reported for many years [24,25]. Resistance to insecticides is linked to a reduction in the sensitivity to drugs due to genetic mutations in the target genes, P-glycoprotein mediated efflux, and the increased metabolic activities of enzymes such as esterases, cytochrome P450, and glutathione S-transferase [26].

Tenutex^®^ is a cutaneous emulsion consisting of a combination of benzyl benzoate (BB) (22.5%) and disulfiram (D) (2.0%) that has been registered in Sweden since 1982 and in Iceland since 1997 for the treatment of scabies and pediculosis (head lice, crab lice) in adults and children [27,28]. Thus, the use of this product is geographically restricted today. Both active substances in Tenutex^®^, benzyl benzoate and disulfiram, have been used individually to treat lice and scabies. Benzyl benzoate is a well-known antiparasitic agent used in many countries to treat lice and scabies, and it is one of the recommended treatments in the European guidelines for the management of scabies [8]. Disulfiram was the active compound in Tenurid, a historical product that was registered in Sweden until 1957 for the treatment of lice and scabies [29]. Disulfiram has been classically used orally as an aldehyde dehydrogenase inhibitor to support alcoholism dishabituation because it produces an increase of acetaldehyde in the blood, leading to patient discomfort, known as the Antabuse reaction [30]. In addition, disulfiram exhibits another mechanism of action, as a chelating agent, with alteration of the cell redox profile and antiproliferative activity, and potential indications as an antimicrobial [31,32] or anticancer [33,34] agent. 

The geographically restricted use of Tenutex^®^ and the fact that the product contains two APIs may be opportunities to avoid or delay resistance to treatment [13]. Although Tenutex^®^ is a well-established drug in Sweden, its biopharmaceutical properties such as skin absorption and its in vitro effects on scabies have yet to be described in the literature, while in vitro effects on human head lice were reported in the early 1980s [35]. 

The aim of this study is to characterize the skin absorption profile of Tenutex^®^ cutaneous emulsion and to study its in vitro efficacy against scabies and human head lice resistant to common insecticides. Since human scabies mites are not easily accessed, tests were performed using sheep scabies (*Psoroptes ovis*) mites as proxy. In addition, the effect of emulsion exposure time on scabies was tested.

## 2. Results and Discussion

### 2.1. Analytical Method Validation

**Figure 1:**
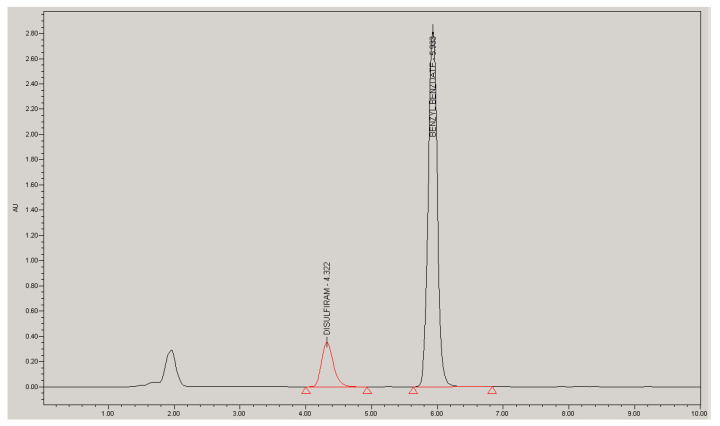
Chromatogram of an analytical standard in the receptor medium at a concentration of 31.7 µg/mL for D and 356.5 µg/mL for BB.

A high-performance liquid chromatography (HPLC) method for the simultaneous analysis of D and BB in samples from the in vitro permeation test was validated according to the ICH Q2 guideline [36]. Figure 1 is a representative chromatogram of an analytical standard in the receptor medium at a concentration of 31.7 µg/mL for D and 356.5 µg/mL for BB. The method’s specificity was confirmed by the lack of an interfering peak at the retention times of both drug standards (diluted in mobile phase) after the injection of a receptor medium blank and excipients of BB/D emulsion (diluted in mobile phase and in the receptor medium). The stability of the drug solution in the HPLC injector was evaluated for 48 h at 25 °C, being the difference in peak area between the two time points; if it is below 2%, then samples are considered stable for this period.

Linearity was assured at the analytical range, with a regression coefficient (r^2^) value higher than 0.990. An acceptable coefficient of variation (CV) of response factor (relationship between response and concentration) was obtained (<10%) considering the high concentration range (3–4 log between the lower and higher concentration). There was a lack of tendency in residual plots. Finally, a significant slope was found for the *p*-value of regression for both drugs (meaning the slope is significantly different from zero), with a nonsignificant *p*-value for the BB intercept (no statistical difference between zero and the intercept) but a significant one with a borderline value of D (Table 1).

Intermediate precision (Table 2 for D and Table 3 for BB) was used as a precision parameter since it had the highest variability (two different analyses on two different days), obtaining global mean values of 104.60% and 95.87% with variation coefficients of 2.61% and 4.01% for D and BB, respectively. Considering a threshold CV value of 6.71%, the method is deemed precise. 

To calculate the accuracy, results from analysis 1 on day 1 were used (Table 2 and Table 3). Global mean values of 103.40% for D and 95.60% for BB, with CV = 2.25% and 3.47% for D and BB, were obtained. Levene’s test was carried out to confirm that no statistical differences were found between the concentration levels (*p* > 0.05 for D and BB), and the confidence interval of global percentage recovery was, in both cases, within the 90.0–110.0% range, showing that the method is accurate between the assayed concentration values. 

Finally, the limit of quantification (LOQ) value was established at a concentration of 0.080 μg/mL for D and 0.8997 μg/mL for BB. 

The analytical method could be considered successfully validated for the analysis of both drug substances in permeation experiments.

### 2.2. Human Skin Permeation

**Figure 2:**
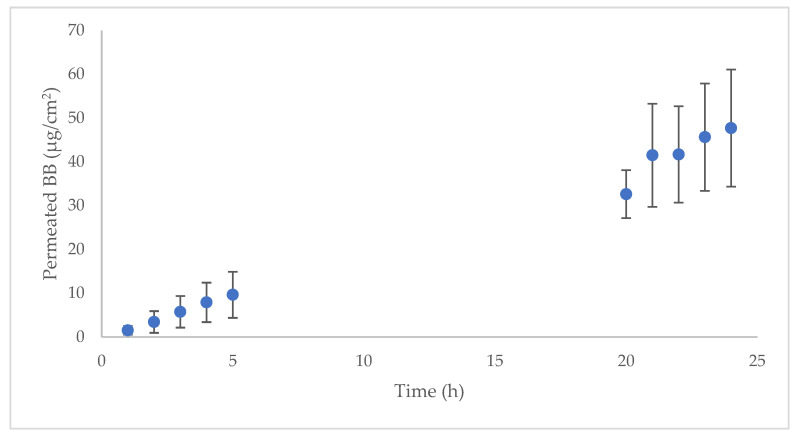
Permeation profile of BB in human skin (*n* = 9) after administration of 3.65 mg/cm^2^ emulsion.

An ex vivo permeation experiment with dermatomed human skin (two different skin donors) was used to characterize the skin absorption profile of D and BB in vertical diffusion Franz cells. Before and after the experiment, the skin integrity was evaluated by measuring the transepidermal water loss (TEWL) value, in all samples and at both testing times; a value below 15 g/m^2^h indicates that the skin maintains integrity. The drug dose evaluated corresponds to the prescribed therapeutic dose (approximately 3.65 mg/cm^2^). According to the technical information available on Tenutex^®^, 60 g of the product should be applied to the entire body surface except the head, which is approximately 16,340 cm^2^ [37]. The results are shown in Figure 2. Only BB was able to penetrate the skin; D was not detected in the chromatogram in any replicate at any time. The permeation parameters are listed in Table 4.

BB had a very good permeation profile, achieving steady state within the first hour after administration (lag time = 0.78 h). This skin absorption is probably caused by the favorable physicochemical properties of the compound, according to the Lipinski rule of five [38]: low molecular weight (212.24 Da), logP value 3.9, low melting point (21 °C), and fewer than five hydrogen bond donors and acceptors [39]. The high diffusion coefficient (Dif) revealed that drug diffusivity is the main absorption mechanism and confirmed the permeability of BB, probably due to its physicochemical properties. The partitioning parameter (P) of the API between the emulsion and the skin surface had a lower contribution to the skin absorption. As previously described, BB has been classically used as a scabicide and was reported to have a good safety profile in its clinical use [5]. In addition, BB is a common ingredient in fragrances, and its safety was previously evaluated [40] and confirmed. Two previous studies [41,42] studied the BB transdermal absorption (in a set of experiments to evaluate the transdermal absorption of compounds used in fragrances), but none of them studied the percutaneous kinetics or the transdermal flux. In addition, the different methodological approaches (dose applied, different vehicles, the use of occlusive material, in vitro or in vivo studies, etc.) in each study make it difficult to compare the results. Although D had good potential permeability, a molecular weight of 296 Da, log P of 3.88, melting point under 200 °C (71.5 °C), and fewer than five hydrogen bond donors and acceptors [43], there was no peak observed in any of the permeation samples’ chromatograms. This may be due to the relatively low concentration of D in the emulsion (2%) compared with 22.5% BB. Considering that D could cause several adverse effects if it is systemically available after oral administration (such as dermatological, neurological, hepatic, gastrointestinal, cardiac, and psychiatric events), mainly due to the inhibition of aldehyde dehydrogenase and dopamine beta hydroxylase [44], the lack of permeability is an advantageous property in terms of treatment safety. D would only be available on the outermost skin layer to develop its antiparasitic effect.

### 2.3. In Vitro Cytotoxicity Evaluation

**Figure 3:**
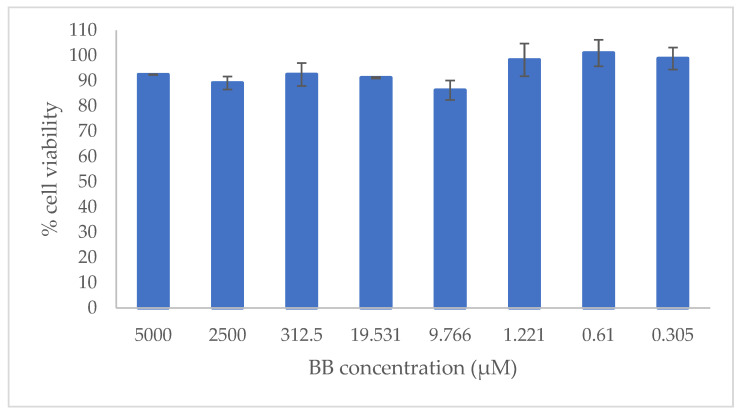
Cell viability 72 h posttreatment with BB. Results show the mean value ± standard deviation (SE).

According to the human skin permeation results, BB was the only component that permeated. The first cells that would come into contact with BB would be skin keratinocytes, so cytotoxicity was tested in human-transformed keratinocyte (HEK001) cells. The concentration tested ranged between 0.305 and 5000 µM, which correspond to 0.647 and 1060 µg/mL, respectively. Seventy-two hours posttreatment, BB had no cytotoxic effect at any concentration (Figure 3).

In the range of concentrations tested, the cell viability was over 85%, with low variability (CV = 5.58%). Luminometric assay determined the number of viable cells in culture based on quantitation of the ATP present, which signals the presence of metabolically active cells [45].

### 2.4. In Vitro and Ex Vivo Efficacy against Scabies

Tenutex^®^, consisting of a BB/D emulsion (22.5% BB/2.0% D), was tested for acaricidal activity in sheep scabies mites in vitro, by exposing the mites directly to the drug emulsion, and via an indirect assay using skin sections excised from sheep. Thus, excised skin sections from sheep were exposed to the emulsion for different lengths of time and then washed off, followed by placing the mites on the skin sections; after incubation, the acaricidal activity was assessed. These dose schemes were chosen to test if shorter product application times were viable, as the summary of product characteristics (SmPC) for Tenutex^®^ stated that the emulsion should be applied to the skin for 24 h before rising it off [27]. Historically, few studies describe in vitro tests carried out to evaluate the efficacy of scabicides, and those that do used the direct in vitro contact method to evaluate the sensitivity for antiparasitic drugs [46,47,48,49]. This method is relatively easy to perform but might not show the treatment efficacy on real application. In this case the product should be at an effective concentration in the most superficial skin layer for drug release and penetration into the skin horny layer. Therefore, the ex vivo assay described in this article was considered to better mimic exposure to the drug after treatment in humans than the direct in vitro test on scabies mites, and it was therefore used to compare with the current prescribed treatment of Tenutex^®^.

It is not possible to culture mites; thus, it is necessary to obtain them from infected humans or animals (pigs are usually employed [48,49]).

In vitro study. The mites exposed to the negative control were unaffected, with a 100% survival rate 24 h postexposure. Mites exposed to the test product showed mortality rates of 100% at 1 h in both the thin- and thick-layer treatment. BB/D emulsion combination has not previously been studied using this model, but other authors described quick scabicide activity with 25% BB product following 3 h of direct contact [46]. In our case, the BB/D emulsion resulted in 100% mortality already after 1 h (Table 5).

Ex vivo study. The efficacy of the Tenutex^®^ emulsion after a 24-h exposure was demonstrated based on clinical evidence [28]. This long period of time with no possibility of rinsing might be inconvenient for the patient. In addition, sweat could remove some of the product. To test if a reduced exposure time is sufficient to obtain the desired efficacy, the pharmaceutical product was washed from the ex vivo skin surface after 10 min, 60 min, or 6 h after application.

**Figure 4:**
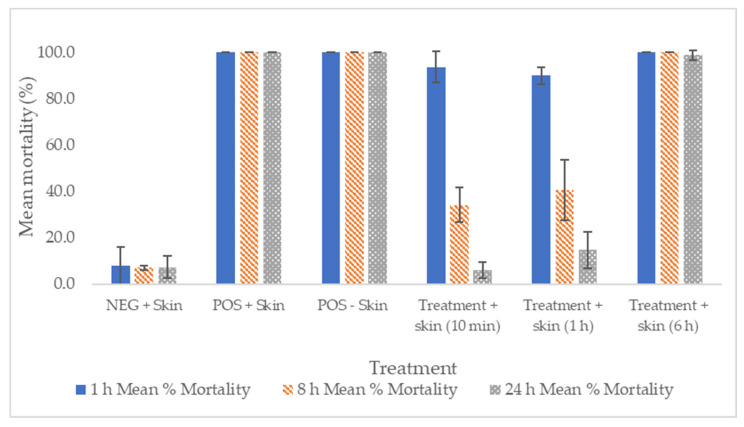
Mean (±S.E.) mortality/unresponsiveness of *P. ovis* mites exposed to the test compound in vitro (‘–skin’) or ex vivo (‘+skin’), expressed as a percentage (%) of mites exposed (*n* = 3 replicates per treatment; *n* = 2 replicates per control). ‘NEG + skin’ = negative control on the skin section (*n* = 47–51 mites per replicate); ‘POS + Skin’ = positive control on skin section (*n* = 50–55 mites per replicate); ‘POS – Skin’ = positive control in Petri dish only (*n* = 50–53 mites per replicate); ‘Treated + Skin (10 min)’= test compound on skin washed after 10 min (*n* = 47–52 mites per replicate); ‘Treated + Skin (1 h)’ = test compound on skin washed after 60 min (*n* = 46–52 mites per replicate); ‘Treated + Skin (6 h)’ = test compound on skin washed after 6 h (*n* = 46–56 mites per replicate).

Skin mites exposed to the test product for 10 min before washing showed an initially high level of mean mortality/unresponsiveness of 93.6% 1 h postexposure. However, mites showed recovery at 8 h and 24 h postexposure (34.2% and 5.8% mean mortality/unresponsiveness, respectively). The final 24-h mean mite mortality for the 10-min treatment was not significantly different to the mean mite mortality of the negative control (*p* = 1.000). A similar trend was observed in mites that had been exposed to skin sections that were treated for 60 min before washing. A mean mortality/unresponsiveness of 89.9% was observed 1 h postexposure, followed by 40.6% and 14.7% mean mortality/unresponsiveness at 8 and 24 h, respectively. The final 24-h mean mite mortality for the 1-h treatment was not significantly different from the mean mite mortality of the negative control (x^2^ = 0.410, df = 1, *p* = 0.529) (Figure 4).

Mites exposed to skin sections treated for 6 h prior to washing showed 100% mean mortality/unresponsiveness at 1 h and 8 h postexposure and 98.8% mean mortality/unresponsiveness at 24 h postexposure. The final 24-h mean mite mortality for the 6-h treatment was significantly different from the mean mite mortality of the negative control (x^2^ = 77.687, df = 1, *p* < 0.001) (Figure 4).

The negative control (on skin section) showed mean mortalities/levels of unresponsiveness of 7.9%, 7.0%, 7.3%, and 8.3% at 1 h, 8 h, 24 h and 30 h postexposure, respectively. The positive control on the skin showed 100% mean mortality/unresponsiveness at all time points (Figure 4).

Based on the results, it is hypothesized that after 6 h of contact, the APIs are absorbed from the emulsion to the skin surface. According to the skin permeation experiments, disulfiram did not cross the skin, and it probably would be restricted to the outermost skin layers, where mites usually reside. In this region, both APIs (disulfiram and benzyl benzoate) would arrive at concentrations that allow the complete mortality of the parasite. The proteomic profile of *Sarcoptes scabiei* was recently published [50], showing several enzymes that disulfiram could interact with, such as glutathione S-transferase [51], ABC transporters [52], and different dehydrogenases [53]. These enzymes are essential for the redox status and the mitochondrial respiratory activity. ABC transporters in addition have a crucial role in the detoxification of xenobiotics, and their inhibition could increase both drug concentrations inside the parasite. The enzymatic inhibitory activity of disulfiram together with the neurotoxic effect of benzyl benzoate are probably responsible for the strong anti-scabies activity of Tenutex^®^. Mites may need a minimum contact of 6 h with the product on the skin in order for us to obtain complete mortality. This potential new dosing scheme could possibly be evaluated in a future clinical trial, but it is a promising result and could facilitate patient compliance and improve patient comfort. For example, patients could apply the product at night before going to sleep, and the following morning, they could wash off the residual product by having a shower.

### 2.5. In Vitro Efficacy against Adults and Eggs of the Human BH-RL Strain of Lice, Resistant to Both Permethrin and Malathion

**Figure 5:**
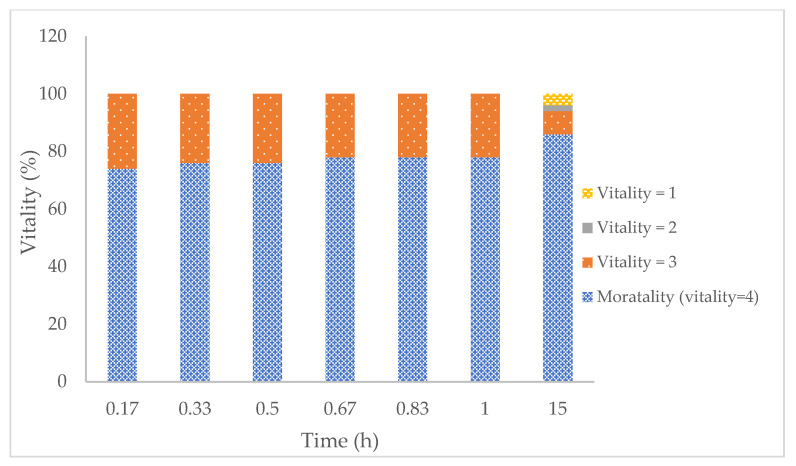
Percentage of young adults observed at a specific vitality classification after 8 h of incubation with Tenutex^®^ emulsion. The treatment was replicated a total of five times, with five males and five females per replication (*n* = 10). Vitality was classified as: 1 (living lice with no change in activity or behavior), 2 (lice with minor changes in vital signs), 3 (lice with major changes in vital signs, i.e., not walking but gut, leg, and/or antennae movement present) and 4 (no vital signs at all).

**Figure 6:**
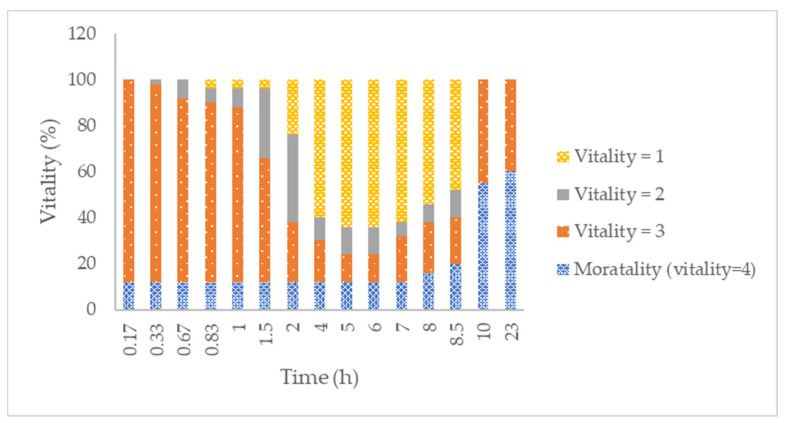
Percentage of young adults observed at specific vitality classification after 10 min of incubation with Nix^®^. Treatment was replicated a total of five times, with five males and five females per replicate (*n* = 10). Vitality was classified as: 1 (living lice with no change in activity or behavior), 2 (lice with minor changes in vital signs), 3 (lice with major changes in vital signs, i.e., not walking but gut, leg, and/or antennae movement present) and 4 (no vital signs at all).

Adulticidal efficacy: Figure 5 and Figure 6 show the vitality signs of adult head lice after incubation with Tenutex^®^ (BB/D emulsion) and Nix^®^ (1% permethrin emulsion) at different contact times. In both cases, a double resistant (malathion and permethrin) strain of *Pediculus humanis capitis* was used. Following an 8-h incubation, BB/D emulsion resulted in an average mortality response (Figure 5) of 74% 10 min after the rinsing procedure; after 15 h, the value increased to 86% but the difference was not significant (*t*-test, *p* < 0.05).

Regarding the reference product (1% permethrin emulsion, ), the mortality is reduced compared to the test product from the first time points studied. Ten minutes post rinse, a 12% mortality response was obtained, and it remained the same for the following 7 h. After 10 h, there was a significant mortality increase, up to 55% (*p* < 0.05), and after 23 h, the mortality response was 60%—not significant compared to the 10-h response. The vitality result for the permethrin treatment showed a significantly lower pediculicidal effect compared to the BB/D treatment. Other studies have evaluated the activity of other 1% permethrin products on resistant strains of hair lice. Subahar et al. [54] showed a 36.7 % mortality rate after 1 h of direct contact with adult parasites. Gao et al. [55] evaluated lethal times of 95% and 50% for a BR-HL-resistant strain obtaining values of 34.6 and 25.8 h, respectively, similar to the 60% mortality found in our study at 23 h.

Finally, the negative control (distilled water) did not show mortality after 15 h, and the mortality response increased to 2% after 23 h. This was significantly different from the Tenutex^®^ and Nix^®^ results. Under the experimental conditions, the Tenutex^®^ emulsion was significantly more toxic than Nix^®^ in permethrin-resistant hair lice. Both permethrin and benzyl benzoate are considered neurotoxic agents against parasites. The higher efficacy of Tenutex^®^ compared to Nix^®^ could be attributed to the combination with disulfiram, giving a synergistic effect against permethrin-resistant mites. The exact mechanism of action of disulfiram against parasites has not been completely elucidated; however, it has been proposed, and as we hypothesized earlier, that it could inhibit several enzymes, such as carbamate kinase, triosephosphate isomerase, ubiquitin-proteosome activity, and aldehyde dehydrogenase, which could mediate the toxicity for Leishmania, Giardia, and Trypanosoma spp., among other parasites [56].

Ovicidal efficacy: Table 6 shows the cumulative hatch rate after treatment with Tenutex^®^ and the negative control (distilled water (ddH_2_O)). Exposure to the test product for 1 h was not enough to obtain an adequate mortality rate (around 14–15% hatch after five days of follow-up). However, a single exposure to Tenutex^®^ for 8 h produced a cumulative hatch rate of 0.2% (corresponding to only one hatch). Finally, exposure to the test product for 24 h achieved complete egg eradication. The egg hatch of the negative control was higher than 98% after exposure to water for 1 or 8 h. The survival of the negative control exposed for 24 h was 87%. Kyong Sup Yoon et al. [57] also tested the hatchability of a BR-HL-resistant strain under normal conditions (not-treated) and found similar values (87.1%) compared to our control (Table 6). In addition, they tested 1% permethrin products over permethrin and DDT double-resistant head lice (SF-HL strain) observing a hatchability of 75%. Jorg Heukelbach et al. [58] found about 60% ovicidal activity of permethrin 1% shampoo for the BR-HL strain and about 95% activity with dimethicone product, similar to our findings with BB/D emulsion.

## 3. Materials and Methods

### 3.1. Materials

The Tenutex^®^ emulsion, placebo, and drug substances (disulfiram and benzyl benzoate) were kindly gifted by Bioglan AB (Malmö, Sweden). Standard disulfiram and benzyl benzoate (Sigma-Aldrich, Burlington, MA, USA) were used for validation of the analytical method. Methanol and KH_2_PO_4_ (Scharlab, S.L., Sentmenat, Spain) were used for the mobile phase. Phosphate-buffered saline (PBS) (Sigma-Aldrich, Burlington, MA, USA) and hydroxypropyl–beta-cyclodextrin (HPCD) (Pracofar, S.L., Martorell, Spain) were used for the preparation of the receptor medium. Dimethyl sulfoxide (DMSO) (Sigma-Aldrich, Burlington, MA, USA), Gibco keratinocyte medium with L-glutamine, and epidermal growth factor (EGF) (Thermo Fisher Scientific, Barcelona, Spain) were used for the cytotoxic evaluation.

### 3.2. High-Performance Liquid Chromatography Quantification and Validation of Disulfiram and Benzyl Benzoate

The simultaneous quantification of disulfiram and benzyl benzoate was carried out with HPLC-UV equipment (Water Alliance 2695, Cerdanyola del Vallès, Spain). Mobile phase (KH_2_PO_4_ 1.7 mg/mL, pH 5.5: methanol (30:70 *v*/*v*)) flowed isocratically at 1.2 mL/min through a C18 column (4.6 × 150 mm, 3 µm, Thermo Scientific, Barcelona, Spain) kept at 30 °C. The injection volume was 40 µL. The method validation was carried out according to the ICH Q2 guidelines [36]. The analytical range was set from 0.080 to 15.85 µg/mL for D and from 0.0899 to 71.97 µg/mL for BB. The linearity, intermediate precision, and accuracy were studied within this range. In addition, the LOQ and solution stability in the HPLC autosampler were determined.

### 3.3. Ex Vivo Skin Absorption Experiment with Human Skin

Dermatomed human skin (approximately 0.5 mm thick), used in the ex vivo permeation test, was obtained from the abdominal region of two different woman during plastic surgery (Condalab, Madrid, Spain). Written consent was obtained from the skin donors. Permeation studies were performed in vertical Franz diffusion cells (VidraFoc, Barcelona, Spain) with a permeation area of 1.54 cm^2^. BB/D emulsion (Tenutex^®^) was dosed in the donor compartment at an equivalent prescription dose (approximately 3.65 mg/cm^2^). Skin was kept frozen at –20 °C until use (six months maximum storage period). On the day of the experiment, skin was thawed at room temperature and placed between the donor and receptor compartment of Franz cells. A 15% *w*/*w* HPCD solution in PBS (pH 5.5) was used as the receptor medium; to maintain sink conditions during the experiment, the temperature was kept at 32 ± 1 °C, with continuous stirring at 700 rpm. Skin integrity was evaluated at the beginning and end of the experiment via TEWL measurement (Delfin Technologies, Kuopio, Finland). Samples from the receptor compartment (300 µL) were taken at regular time intervals up to 24 h and replenished with the same volume of fresh receptor medium. Samples were analyzed and quantified using the method described in the previous section.

After drug quantification, the following permeation parameters were calculated: the transdermal flux (J, μg/cm^2^h) (Equation (1)), permeability coefficient (Kp, cm/h) (Equation (2)), lag time (tlag, h) (obtained by linear extrapolation of the x-axis of the points at steady state), diffusion parameter (Dif, 1/h) (Equation (3)), and partitioning parameter (P, cm/h^2^) (Equation (4)).

(1) $$J=\frac{dQ}{\left(dT\cdot \;S\;\right)}\;$$

where J is the transdermal flux, dQ is the permeated amount differential, dT is the time differential, and S is the membrane diffusion surface.

(2) $$Kp=\frac{J}{C_{d}}$$

where Cd (μg/mL) is the concentration of the drug in the donor compartment.

(3) $$Dif=\frac{1}{6T_{lag}}$$

(4) $$P=\frac{Kp}{D}\;$$

### 3.4. In Vitro Cytotoxicity Evaluation

Cell treatments (*n* = 6): human-transformed keratinocyte (CRL2404, ATCC, Manassas, VA, USA) cells were seeded in 96-well plates at 10,000 cells/well in 50 µL of medium (keratinocyte serum-free medium, supplemented with 2 mM L-glutamine and 5 ng/mL Epidermal Growth Factor (hEGF)). The cells were incubated at 37 °C in a 5% CO_2_, 95% air-humidified atmosphere for 24 h. After 24 h of incubation, the cells were treated with increasing doses of BB (0.305 to 5000 µM; a stock solution was prepared in DMSO and the final concentration of DMSO in the wells was below 1%). A medium with DMSO at 1%, sodium dodecyl sulfate at 10%, and a cell culture medium were used as the experimental control. The exposure period was 72 h.

At the end of the incubation time, cytotoxicity was evaluated using the CellTiter-Glo^®^ Luminescent Cell Viability Assay (Dojindo Molecular Technologies, Rockville, MD, USA). One hundred microliters of CellTiter-Glo^®^ reagent were added directly to every well plate, cultured in serum-free medium, and incubated for 10 min. The luminescence was measured immediately using a Victor X3 luminometer (Perkin Elmer, Waltham, MA, USA).

### 3.5. In Vitro and Ex Vivo Efficacy against Scabies

To evaluate the in vitro and ex vivo efficacy of BB/D emulsion against scabies, *Psoroptes ovis* mites (sheep scabies) were selected as the model, since the ethical sourcing of human scabies is almost impossible.

One-year-old Scotch Mule lambs (*n* = 3) were infested with *P. ovis* mites and housed indoors for a period of six weeks. After that time, animals were sacrificed via an anesthetic overdose. The study protocol (1258/CC1406) was approved by the London School of Hygiene and Tropical Medicine ethics committee (reference 2020-07A, approval date: 26 May 2020). Skin sections (5 cm × 5 cm) were taken with a scalpel and deposited in a Petri dish at room temperature to harvest live mobile mites. Once obtained, mites were washed with purified water on a 63-µm filter and dried with blotting paper and stored in an Eppendorf tube until the study (within 2 h).

In vitro study. Petri dishes were coated with a thin or thick layer of BB/D emulsion or the placebo. Collected mites were placed on corresponding dishes (20–30 mites per replicate and three replicates per condition) and incubated at 28 °C/75% relative humidity (HR) for 1, 8, and 24 h. After this period, acaricidal activity was assessed via stereomicroscopic examination (mites were considered viable if any movement was observed).

Ex vivo study. Single sheep scab naïve was sacrificed and skin sections (4 cm × 4 cm) were excised with a scalpel and placed in a Petri dish. Adult female mites were obtained from the collected mites previously described. Around 50 *P. ovis* mites were immediately applied to ex vivo skin sections (positive and negative control and test samples) and incubated at 28 °C/75% HR. Two positive controls were used: product in Petri dish (not washed off) with mites applied directly to the product, and product on skin (not washed off) with mites applied directly to the skin. The negative control was the skin with no product and mites applied directly to the skin. In the experimental condition, a thin layer of emulsion was applied to the skin and washed with purified water 10 min, 1 h, and 6 h after exposure. Acaricidal activity was assessed via microscopic examination after 1, 8, and 24 h.

### 3.6. In Vitro Efficacy against Permethrin-Resistant Head Lice and Its Eggs

To evaluate the efficacy of the BB/D emulsion against lice adults and eggs, permethrin- and malathion-resistant human head lice (*Pediculus humanis capitis*, BR-HL strain) were used [55,57].

Blood-fed adults, half or fully engorged (*n* = 10; 5 males and 5 females), were transferred to hair tufts. Five milliliters of BB/D emulsion or distilled water (used as a negative control) were gently rubbed for 60 s and incubated at room temperature for 8 h. Then, the tuft was washed with a commercial shampoo diluted at 5% (*w*/*w*) in distilled water for 60 s to remove the excess product and subsequently rinsed carefully with distilled water so as not to detach the adult lice. Finally, the hair was allowed to dry on filter paper and lice were examined for viability at different time points. A total of five replicates were carried out for each condition. Lice were classified as: 1 (living lice with no change in activity or behavior), 2 (lice with minor changes in vital signs), 3 (lice with major changes in vital signs, i.e., not walking but gut, leg, and/or antennae movement present) and 4 (no vital signs at all). To validate that the lice were resistant to permethrin, the same study was conducted with a permethrin 1% commercial product (Nix ^®^) with observation for up to 23 h.

Eggs were obtained from female adults that were placed on a tuft of human hair and left until approximately 50 eggs were laid per hair tuft. Then the adults were removed. BB/D emulsion or distilled water (5 mL) was applied as previously described in the adult experiment. Incubation was carried out at room temperature in a dark fume hood for 1, 8, and 24 h. Then, the hair tuft with the eggs was washed and rinsed as previously described. Dried tufts with the eggs were placed in a Petri dish and incubated at 31 °C and 70–80% relative humidity. Egg viability (%hatchability) was evaluated for 11 days, according to Equation (5). A total of three replicates was carried out.

% Hatchability = Number eggs hatched/total eggs oviposited × 100(5)

### 3.7. Statistical Analysis 

To assess the in vitro efficacy against scabies, a survival curve analysis was performed on data using STATA (version 15.1) with a log rank (Mantel–Cox) test applied to compare survival curves.

To test ex vivo efficacy, a Chi-square test (or Fisher’s exact test) was performed in R studio to compare the mean proportion of the negative control against each washing interval treatment after conducting a test of homogeneity between replicates.

## 4. Conclusions

An analytical method validation was successfully developed for the quantification of D and BB permeation studies. The permeation profiles on dermatomed human skin revealed fast permeation of BB and non-permeation of D. Moreover, the cytotoxicity assay showed the noncytotoxic effect of BB and confirmed the safe topical use of this compound, together with the low adverse effects reported in the literature for this treatment.

The results of in vitro and ex vivo efficacy demonstrated that Tenutex^®^ had a strong knockdown effect on *P. ovis* within 1 h of exposure on all the skin sections treated. However, on the skin sections treated for the shortest periods of time, mite recovery was observed over time. In contrast, mites exposed to the skin sections treated for 6 h before washing showed almost 100% mean mortality/unresponsiveness at all time points. This fact could potentially improve patient compliance, reducing the exposure contact time, compared with the dose scheme described in the current SmPC (24 h of contact time).

The in vitro efficacy studies against the adults and eggs of human lice showed that Tenutex^®^ was substantially adulticidal on the permethrin-resistant BR-HL strain following an 8-h exposure. Moreover, the product was 100% ovicidal following a 24-h exposure. The BB/D emulsion killed almost all of the lice eggs after an 8-h exposure (99.8%).

This study has provided more information on the efficacy and biopharmaceutical properties of Tenutex^®^, a cutaneous emulsion containing a fixed-dose combination of the APIs benzyl benzoate and disulfiram and used for the topical treatment of human scabies and lice. The balance of properties of this unique combination makes Tenutex^®^ a good treatment option, especially in situations where resistance to other drugs has been encountered.

## Figures and Tables

**Table 1:** Regression line for D and BB, range, slope, intercept, coefficient of variation (CV) of response factor, residual plot tendency, and regression coefficient. The distribution was assessed via an equal variance test, Levene’s test (α = 0.05).

Parameter		D	BB	Specification
Range		0.08–15.70 µg/mL	0.90–182.70 µg/mL	-
Linearity	Slope	137,322.71 (*p* < 0.05)	84,127.02 (*p* < 0.05)	*p* < 0.05
	Intercept	–7049.71 (*p* = 0.04)	–16,389.67 (*p* > 0.05)	*p* > 0.05
R^2^		0.99980	0.99997	>0.990
CV response factor		8.71%	0.71%	<10%
Homoskedasticity (Levene’s test)		*p* = 0.31	*p* = 0.83	*p* > 0.05
Residual plot		No tendency	No tendency	No tendency

**Table 2:** Results of intermediate precision for disulfiram at three different concentration levels. Mean and CV values are reported.

Concentration Level	Sample ID	Disulfiram		
0.08 μg/mL	Analyst 1 day 1 Analyst 2 day 2	Mean: 101.20% CV = 2.52% Mean:105.00% CV = 2.54%	Mean: 103.12% CV = 3.03%	Mean: 104.60% CV: 2.61%
0.38 μg/mL	Analyst 1 day 1	Mean:104.20% CV = 1.92%	Mean: 103.95% CV = 1.37%	
	Analyst 2 day 2	Mean: 103.70% CV = 0.94%		
6.35 μg/mL	Analyst 1 day 1	Mean: 104.80% CV = 0.27%	Mean: 106.62% CV = 2.19%	
	Analyst 2 day 2	Mean: 108.40% CV = 1.84%		

**Table 3:** Results of intermediate precision for benzyl benzoate.

Concentration Level	Sample Id.	Benzyl Benzoate		
0.89 μg/mL	Analyst 1 day 1 Analyst 2 day 2	Mean: 91.70% CV = 1.98% Mean: 90.30% CV = 1.81%	Mean: 91.02% CV = 1.88%	Mean: 95.80% CV = 4.01%
3.57 μg/mL	Analyst 1 day 1 Analyst 2 day 2	Mean: 96.30% CV = 0.37% Mean: 97.60% CV = 0.49%	Mean: 98.90% CV = 0.59%	
71.42 μg/mL	Analyst 1 day 1	Mean: 104.80% CV = 0.27%	Mean: 100.10% CV = 0.86%	
	Analyst 2 day 2	Mean: 108.40% CV = 1.84%		

**Table 4:** Permeation parameters of benzyl benzoate in human skin.

	Mean	SD
Jsup (µg/hcm^2^)	1.3014	0.3366
R^2^	0.9963	0.0067
Kp (cm/h)	5.78 × 10^−6^	1.50 × 10^−6^
Tlag (h)	0.7755	0.7059
P (cm/h^2^)	3.5978 × 10^−5^	1.3657 × 10^−5^
Dif (1/h)	0.1252	0.0183

**Table 5:** Mean percentage mortality across three replicates following exposure to Tenutex^®^ emulsion or placebo. With incubation at 28 °C with 75% relative humidity for 0, 1, 8, and 24 h.

Test Solution	Mean % Mortality			
	0 h	1 h	8 h	24 h
Placebo—thin layer (*n* = 91 mites)	0	0	0	0
Placebo—thick layer (*n* = 88 mites)	0	0	0	0
Tenutex emulsion—thin layer (*n* = 91 mites)	0	100	100	100
Tenutex emulsion—thick layer (*n* = 90 mites)	0	100	100	100

**Table 6:** Average % hatchability with standard deviation of eggs for three replicates following tuft submersion in either Tenutex^®^ or distilled water (the negative control) for 1, 8, or 24.

	Average % Hatchability ± SD
Tenutex, 1 h	15 ± 18
ddH_2_O, 1 h	96 ± 2
Tenutex, 8 h	0.2 ± 0.4
ddH_2_O, 8 h	98 ± 1
Tenutex, 24 h	0 ± 0
ddH_2_O, 24 h	87 ± 4

## Data Availability

The data presented in this study are available on request from the corresponding author. The data are not publicly available due to intellectual properties restrictions.
